# Lectures on Diseases of the Lungs and Heart

**Published:** 1836-04-01

**Authors:** 


1. Lectures on the Diseases of the Lungs and Heart. By *Thomas Davies*, M.D. Octavo, pp. 512. London, 1835.

2. Traite Clinique des Maladies du Cœur. Par *J. Bouillaud*. 2 Tomes, 8vo. Paris, 1835.

These are the works of two excellent physicians, and most accomplished stethoscopists. The lectures of Dr. Davies have already appeared separately in the Medical Gazette, and they form one of the most valuable contributions which have of late enriched the pages of that periodical. As they are truly and strictly "lectures," addressed to a body of students, and intended merely as a manual of reference, our duty, as reviewers, is necessarily limited to the expressing of our opinion of their general merits. They will be found to contain an exceedingly clear and well digested resumé of information on most of the important topics of thoracic pathology. Each topic is treated succinctly, and with ability, and the practical remarks on the treatment of the various diseases of the heart and lungs are valuable, as coming from a physician who is well known to have devoted much of his time and talents to their minute investigation.

We observe that Dr. Davies reports most favourably of the effects of the antimonial treatment of peripneumonia. He commences with doses of a third of a grain (adding occasionally a few dops of tinct. opii, or syr. papav.) every hour, gradually increasing the quantity by a third of a grain every two hours, until he reaches two grains. It is useful in all the stages of the disease, but especially in the first, that of engorgement. In old and cachectic persons, who cannot bear bleeding well, Dr. D. says "he has found the tartar-emetic, carefully administered, produce frequently the most astonishing curative effects;" and, he adds, "I have frequently left patients, under the impression that their recovery was almost impossible, and yet have found that the continuous use of this medicament has snatched them from the jaws of death."

With respect to the comparative efficacy of mercury and tartar-emetic, he appears to give the preference to the latter, especially in the earlier stages of peripneumonia, and to the former, when the structure of the lungs has become, or is becoming, hepatised; and he concludes his remarks by stating, "that the tartar-emetic seems to act most energetically upon inflammations of the parenchymatous tissues of the organs, and mercury upon those of the serous membranes."

In the treatment of angina pectoris, Dr. Davies praises in high terms the application of a belladonna plaster, of the size of the hand, formed of one part of the extract with four of soap plaster, over the region of the heart.

He records a curious case of patency of the foramen ovale in a youth, eighteen years of age, in whom he had every reason to believe that the lesion was not congenital, but had been caused by a rupture of the septum, brought on by violent exercise at cricket, six years before his decease. Here we must stop our notice of Dr. Davies' lectures.

The treatise of M. Bouillaud lays claim to much higher pretensions. It is evidently the production of a most able and vigorous mind, which is accustomed to think, and reason, and determine for itself. Hence there is much originality of research and speculation pervading every part, and the novel and striking lights in which the opinions and discoveries of others are placed, add greatly to the interest of the descriptions. Some of this author's conclusions, indeed, are too rapid and premature, and his assertions every now and then are bold and peremptory ; but, withal, the present work is one of high and standard merit, and, were we asked to point out a favourable specimen of modern French medical literature, we know of none to which we should with greater confidence appeal than to the clinical treatise of Professor Bouillaud. Besides a copious narrative and general description of every cardiac derangement, it contains faithful records of upwards of 200 cases of disease. It is, therefore, quite a magazine of clinical observations, to which all future writers will, we venture to predict, make frequent reference.

After giving a most ample history of the anatomy of the heart, he proceeds to examine the various theories which have been proposed to account for the double or tic-tac sound, which attends the actions of that organ. He decides in favour of the theory of M. Rouanet, who attributes the first sound to the sudden closing and apposition of the auriculo-ventricular valves, and the second to the closing and apposition of the semilunar valves of the aorta and of the pulmonary artery. M. Bouillaud suggests that the falling back, or "abaissement," of the one or open set of valves, at the time while the other is closing, may possibly contribute to the production of the sounds in question ; but he admits that they are mainly owing to the instantaneous closing and succussion of the divisions of the valves against each other; of the auriculo-ventricular during the systole, and of the semilunar during the diastole of the ventricles.

Having discussed, at great length, the anatomical and physiological portions of his work, the various morbid conditions of the heart and its investing membrane, from simple inflammatory or nervous affections to congenital malformations, are then amply reviewed and illustrated. The details will be found full of interest and instruction, and had our limits permitted us to enlarge, we should have gladly devoted a long article to their examination. But, at present, we cannot afford the requisite space. We shall, therefore, select only one subject for a few remarks, and that subject will be the inflammation of the internal lining membrane of the cavities of the heart, or endocardium, as our author calls it. This disease, ENDOCARDITIS, has been more minutely investigated by M. Bouillaud than by any other writer. It was he who first gave it a name (which, by the bye, has not been yet recognized by English physicians), and established it as a legitimate member of the nosological code. Most of its effects, or sequelæ, as thickening, vegetations, and ossifications of the valves, contraction of the orifices, hypertrophy of the ventricles, polypi, &c. have been described by most authors as idiopathic diseases. M. B. traces them, in almost every case, to a precursory inflammation, acute or chronic, of the endocardium, and he points out the close analogy which may be traced between inflammation of this membrane and inflammation of the pericardium. Many of their symptoms are alike—the post-mortem appearances are very similar—the inducing causes are the same, and the same treatment is applicable to both. M. B. divides the history of endocarditis into three periods or stages—the period of congestion, softening, suppuration, and ulceration; the period of the organization of the secreted products, or of a portion of the fibrinous coagula; and the period of cartilaginous, osseous, or calcareous induration of the endocardium in general, and of the valves in particular, with or without contraction of the orifices of the heart.

The anatomical characters of the first period or stage engage our attention first.

An unusual redness of the endocardium is discovered in an immense majority of the cases. The shade of colour varies from a pink to a scarlet, purplish, or even brown hue. The redness may be partial or general; very often it is confined to the surface of the valves, and, when it is more extensively diffused, it is always of a deeper colour on the valvular portions of the endocardium than elsewhere. M. Bouillaud states that this redness is owing, not to any capillary congestion, but rather to a "sanguineous dyeing" of the lining membrane of the heart. [This statement surprises us.] It does not extend beneath this membrane. It is not removable by simple washing; but maceration, continued for some time, will cause it to disappear.

M. B. is well aware that many pathologists regard the redness of the endocardium, of whatever hue it may be, and in all cases, as the result of cadaverous imbibition; and he admits that the appearance is very commonly seen in the corpses of patients who have died of typhus or other diseases, in which the mass of the blood has become unusually fluid, and otherwise changed, more especially if the process of putrefaction has at all commenced when the body is examined. But, when we discover this redness in a body of a patient recently dead, and who during life had laboured under the symptoms of endocarditis (to be hereafter described), there is reason, M. B. thinks, to attribute it to inflammatory action. If the redness be associated with any swelling, puffiness, or thickening of the affected surface; if there be any traces of purulent or pseudo-membranous deposit; if there be any adherent, discoloured, fibro-albuminous coagula; or lastly, if there be co-existent redness of the internal surface of any bloodvessels, which, it was known, had suffered from inflammation, (as in cases of phlebitis,) there is strong reason for believing that the phenomenon, we are now alluding to, is the result of a vital change.

Whenever endocarditis has lasted for some time, the internal membrane of the heart is very generally found somewhat thickened. This appearance is always most conspicuous on and around the valves. The membrane may be, at the same time, more easily lacerable than usual, and also more readily detached from the subjacent cellular texture. Sometimes minute erosions, or incipient ulcerations of the endocardium are visible at different points.

The early vestiges of any purulent or pseudo-membranous deposit on the endocardium are necessarily rendered indistinct, in consequence of the secreting surface being constantly washed by the current of the circulating blood. The pus, when it does exist, is found sometimes in the meshes of the columnæ carneæ, at other times in the centre of adherent coagula. Detached portions, or specks of lymph, are not unfrequently seen on the surface of the valves, or of their tendons, presenting the appearance of rounded granulations or vegetations.

These pathological changes of the endocardium are very generally accompanied with certain changes of the blood itself, within the affected cavities. Coagula, varying in size and number, are found adhering at different points. These coagula are whitish, elastic, glutinous, adherent to the walls of the cardiac cavities, twisted round and entangled with the chordæ tendineæ and columnæ carneæ. They are, in some degree, organized, and may be aptly compared to pseudo-membranous deposits. In some of them we observe minute red points and lines, which are, in fact, the rudiments of bloodvessels. These coagula, the result of endocarditic disease, are very frequently continued for some way into the large vessels. They are more common, and generally also more voluminous, in the right than in the left cavities. Usually they are most strongly adherent to and around the free edges of the valves. M. Bouillaud is of opinion that the vegetations or granulations of the valves, an appearance of so frequent occurrence, very often originate in the deposition of these small fibrinous masses.

The second period or stage of endocarditis is characterised by the thickening of the inflamed tissue, and by the organization of the lymph or fibrine, which had been secreted during the first stage. Hence the origin of the vegetations, as alluded to above, which are so frequently found on the valves, and on their tendinous cords—of the adhesions of portions of the valves to each other, or to the surface of the ventricles, and of the milky-looking depositions of organized lymph, in patches of greater or less extent, which are often seen on the surface of the cardiac cavities.

The anatomical characters of the third period are the cartilaginous, osseous, or calcareous formations, which are so generally met with in all protracted cases of cardiac disease, affecting the valves, bloodvessels, or substance of the heart itself. The numerous lesions resulting from these changes have been fully illustrated by all the late authors on the diseases of the heart, and by none more completely than by M. Bouillaud in the present treatise. He alludes particularly to the contraction of the valvular orifices, which is induced by the gradual induration and ossification of their structure, and to the subsequent changes in the tissue of the heart itself, and of other distant organs, which are so frequently consequent on the valvular disease. Thus, hypertrophic enlargement of the ventricles is an almost invariable sequence of prolonged endocarditis ; and M. Bouillaud is of opinion, that many of the lesions of the pericardium are secondary, and consecutive to those of the endocardium, in consequence of the intimate sympathy between these two analogous membranes. Of thirty-four cases of endocarditis reported, seventeen presented distinct traces of a co-existent pericarditic disease. The distention of the venous system, more especially of the veins of the internal viscera, and the superinduced tendency to watery exhalation into the serous cavities, and into the general cellular substance, are well-known sequelæ of cardiac disease.

M. B. devotes several pages to answering the objections which may be, and, indeed, have been urged against his hypothesis, that most, if not all, of the morbid lesions of the valves and other internal parts of the heart's structure are attributable to endocarditic disease. His reasoning, and the facts adduced in support of it, well deserve the study of the physician. The following extract from Andral's Clin. Med. tom. 1ier, affords powerful testimony to the correctness of our author's views.

" If the inflammatiou of the internal membrane of the heart passes into the chronic state, the membrane becomes gradually more and more thickened, and especially where it is doubled upon itself to form the different valves. Not only is this membrane thickened, but it also becomes the seat of vegetations, and of other kinds of degeneration. The fibrous tissue likewise, which is invested by the membrane, is usually indurated; and at these parts, as well as at every other, where it is affected with inflammation, this tissue shews a tendency to a cartilaginous or bony change. The effect of all these various alterations is to narrow the orifices of the heart, to obstruct the free play of the valves, and, consequently, to distress the course of the blood. Thus, then, a great number of the contractions of the different cardiac orifices, induced either by vegetations on the surface of the investing membrane, or by cartilaginous or osseous concretions situated under it, are attributable primarily to acute or chronic endocarditis, as their 'point de depart.' "

So much for the morbid anatomy of endocarditis. We shall now briefly detail the symptoms which indicate the disease during life.

It must be acknowledged that, in a large number of cases, the symptoms are very indistinct and unsatisfactory. There is usually more or less of pain in the region of the heart, and this is accompanied with a sense of distressing oppression, anxiety, tendency to leipothymia, and with general malaise of the system. The pulsations of the heart are stronger and more diffused than in health, and they are apt to be tumultuous: at times they are attended with a most distinct vibratory tremor of the thoracic parietes. A bruit de soufflet, more or less loud, may almost always de detected; and, when the action of the heart is violent, each period of the ventricular systole is accompanied with a "tintement mètallique." The pulse at the wrist is usually rapid, irregular, and unequal. The general system very generally sympathises with the cardiac disease, and pyrexia, more or less distinctly marked, is present during the early stages of endocarditis. When the disease has continued for a length of time, and especially when the valves are much affected, or when there are considerable coagula within any of the cavities, the venous circulation exhibits various signs of embarrassment. The same may be said of the effects of endocarditis on respiration.

M. B. acknowledges that the symptoms which he has enumerated as indicative of endocarditis, during its earlier stages at least, are by no means pathognomonic of that disease, and that they appertain almost as constantly to pericarditis. Fortunately, no practical evil can flow from this obscurity; and, moreover, it is to be remembered, that the outer and inner investing membranes of the heart very generally suffer together in disease. When the organic lesions of the valves and other parts of the heart's structure become more decided and serious, the symptoms are usually sufficiently expressive of the mischief that is going on.

With respect to the etiology of endocarditis, M. Bouillaud states, that an extensive experience has now convinced him that, (as is the case with pericarditis,) rheumatic, or, as it ought to be called, arthritic inflammation is by far the most frequent cause of the disease. He strongly urges on the attention of his readers the reflection, that it does not require any metastasis of the articular disease to the heart to excite mischief there; but that, very often, the cardiac inflammation is developed at the very commencement of the attack, and is co-existent with the affection of the joints, during its entire progress. The infinite importance, therefore, of counteracting and arresting arthritic inflammation, wherever it may exist, and however partial and trifling it may seem, must be abundantly obvious. M. Bouillaud, more than any other author, has the merit of having illustrated the practical position, that a most intimate relationship exists between the inflammatory affections of the synovial and other apparatus of joints, and those of the outer and inner investing membranes of the heart.

M. Bouillaud appends some excellent remarks on the appropriate treatment of endocarditis, and of the various organic lesions which it so often induces. Small and repeated bleedings, especially from the cardiac region, either by means of cupping, or of leeches, the frequent application of blisters, the internal and external use of digitalis, the employment of colchicum, hydriodate of potass, and of other saline diuretics, along with absolute repose and restriction to a very mild unirritating diet; these are the means from which the patient will derive the greatest benefit.

